# Sex Differences in the Outcomes of Cryoablation for Atrial Fibrillation

**DOI:** 10.3389/fcvm.2022.893553

**Published:** 2022-05-18

**Authors:** Alexis Hermida, Jacqueline Burtin, Maciej Kubala, Floriane Fay, Pierre-Marc Lallemand, Otilia Buiciuc, Audrey Lieu, Mustafa Zaitouni, Christophe Beyls, Jean-Sylvain Hermida

**Affiliations:** ^1^Cardiac Arrhythmia Service, Amiens-Picardie University Hospital, Amiens, France; ^2^Clinique de l'Europe, Amiens, France

**Keywords:** female, atrial fibrillation, paroxysmal atrial fibrillation (PAF), persistent atrial fibrillation, cryoballoon ablation

## Abstract

**Background:**

The literature data on the outcomes of radiofrequency catheter ablation for atrial fibrillation (AF) in women are contradictory.

**Aim:**

To determine and compare the outcomes and complications of cryoballoon pulmonary vein isolation (cryo-PVI) in men vs. women, and to identify predictors of atrial tachyarrhythmia (ATa) recurrence.

**Methods:**

We included all consecutive patients having undergone cryo-PVI for the treatment of symptomatic AF in our center since 2012. Peri-operative complications were documented. All patients were prospectively monitored for the recurrence of ATa, and predictors were assessed.

**Results:**

A total of 733 patients were included (550 men (75%) and 183 (25%) women). Paroxysmal AF was recorded in 112 (61%) female patients and 252 male patients (46%; *p* < 0.001). Female patients were older (*p* < 0.001) and had a greater symptom burden (*p* = 0.04). Female patients were more likely to experience complications (*p* = 0.02). After cryo-PVI for paroxysmal AF, 66% of the female patients and 79% of the male patients were free of ATa at 24 months (*p* = 0.001). Female sex was the only independent predictive factor for ATa recurrence (hazard ratio [95% confidence interval] = 1.87 [1.28; 2.73]; *p* = 0.001). After cryo-PVI for non-paroxysmal AF, 37% of the male patients and 39% of the female patients were free of ATa at 36 months (*p* = 0.73). Female patients were less likely than male patients to undergo repeat ablation after an index cryo-PVI for non-paroxysmal AF (*p* = 0.019).

**Conclusion:**

A single cryo-PVI procedure for paroxysmal AF was significantly less successful in female patients than in male patients. Overall, the complication rate was higher in women than in men.

## Introduction

Although atrial fibrillation (AF) is a serious disease in both sexes (1), some researchers have stated that this condition is a stronger risk factor for cardiovascular disease (2) and death in women than in men (2, 3). However, women undergo ablation less frequently (4–6)—even though they typically have a greater symptom burden (5–12)—and are referred to a specialist later in the course of the disease (13, 14).

Some studies (5, 14–21) [but not others (11, 13, 22–25)] indicate that women have more complications after radiofrequency ablation. Furthermore, some researchers argue that the outcome of radiofrequency ablation is worse in women (11, 14, 19, 20, 26, 27), which might dissuade cardiologists from suggesting this procedure. Conversely, other studies did not evidence sex difference in the outcome of radiofrequency ablation (5, 12, 13, 22–24, 28, 29).

Hence, we sought to determine whether sex differences were observed in a large cohort of patients having undergone cryoballoon pulmonary vein isolation (cryo-PVI) for AF. We also sought to identify predictors of the long-term recurrence of atrial tachyarrhythmia (ATa).

## Method

### Study Design

We included all consecutive patients having undergone cryo-PVI for the treatment of AF since the introduction of second-generation cryoballoon ablation at the Amiens University Hospital (Amiens, France) between September 2012 and July 2019. The patients gave their written informed consent to participation in the study. The study protocol conforms to the ethical guidelines of the 1975 Declaration of Helsinki. In line with the French legislation on studies of routine clinical practice, the study protocol was approved by a hospital committee with competency for studies not requiring approval by an institutional review board. Furthermore, the study database was registered with the French National Data Protection Commission [*Commission nationale de l'informatique et des libertés* (Paris, France); reference: PI2020_843_0080].

The study's primary objective was to compare male vs. female patients with regard to success rates and complication rates after an index cryo-PVI procedure. The secondary objective was to identify the long-term predictors of ATa.

### Study Population

Patients were included if they had undergone cryo-PVI for the treatment of AF documented with a 12-lead ECG. The exclusion criteria were (i) prior left atrial (LA) ablation, (ii) LA tachycardia at the time of cryo-PVI, (iii) age under 18, (iv) loss to follow-up after the cryo-PVI procedure, and (v) a prosthetic heart valve or congenital heart disease. Patients with intracavitary thrombus or a contraindication to anticoagulation (heparin, vitamin K antagonist or direct oral anticoagulant use) were excluded before the cryo-PVI.

Atrial fibrillation was classified according to the 2020 European Society of Cardiology guidelines (30). Persistent AF was defined as AF lasting for more than seven days, including episodes terminated by cardioversion. Patients with long-standing, persistent AF (i.e. continuous AF for a year or more) were also included. The duration of AF was defined as the time interval between the first episode of AF and the cryo-PVI procedure.

The LA area was measured by echocardiography with a four-chamber view. The left ventricular ejection fraction (LVEF) before cryo-PVI was also estimated. The LA volume was obtained from the segmentation of multidetector CT data using AW VolumeShare 5 software (General Electric, Fairfield, CT, USA). The first centimeter of each pulmonary vein and the LA appendage were included in the LA volume. The presence of an accessory vein or a left common trunk was assessed on a CT scan.

Hypertension was defined as repeated in-office or out-of-office systolic blood pressure values ≥ 140 mmHg and/or diastolic blood pressure values ≥ 90 mmHg prior to treatment for hypertension.

### The Cryo-PVI Procedure

Cryoballoon ablations were preceded by at least three weeks of treatment with an oral anticoagulant and then by a contrast-enhanced multislice CT assessment of the left atrium.

Access to the left atrium was gained after fluoroscopy-guided transseptal puncture (Abbott Laboratories, Abbott Park, IL, USA). The sheath was flushed continuously with heparinized saline. Only one four-minute application was performed with a second- or third generation cryoballoon (the 23- or 28-mm Advance balloon, Cryocath, Medtronic, Dublin, Republic of Ireland). An intraluminal catheter (Achieve^TM^, Medtronic) was used for all procedures; it enabled real-time visualization of the arteriovenous block during the cryoapplication. At the end of the applications and then again 15 min later, we checked for a bidirectional block in each of the pulmonary veins by alternately stimulating the Achieve^TM^ catheter and the coronary sinus. This ensured that the pulmonary vein isolation was complete at the end of the procedure, including in patients for whom the block was not visible during the shots. A second cryoapplication was initiated (to achieve a complete block) only when venous conduction persisted.

The shots were stopped when the temperature fell below −60°. During cryoablation over the right pulmonary veins, a quadripolar catheter was used to continuously pace the phrenic nerve adjacent to the superior vena cava. In order to avoid right phrenic nerve palsy, cryo-PVI was discontinued immediately if the diaphragm stopped contracting.

Data collected during the procedure included: the time to effect, the trough temperature during cryo-ablation for each PV, the duration of cryo-ablation for each PV, the cumulative duration of cryo-ablation to the PVs, the number of cryoballoon applications for each PV, the fluoroscopy time, and the fluoroscopy dose.

All adverse events occurring during the procedure or in the month thereafter were recorded.

### Follow-Up

After cryo-PVI, antiarrhythmic and anticoagulant treatments were maintained for at least the three-month blanking period.

Patients underwent follow-up examinations in our institution 3, 6, and 12 months after the cryo-PVI procedure; this included a physical check-up, screening for arrhythmia-related symptoms, a 12-lead ECG, and 24 h Holter monitoring. After the first year, patients were followed up by their cardiologist at least annually, with a 12-lead ECG and 24 h Holter monitoring. The presence of any symptoms after ablation prompted an assessment with a 12-lead ECG and 24 h Holter monitoring. Previously implanted pacemakers or dual-chamber implantable cardioverter defibrillators were checked for atrial high-rate events at each follow-up visit.

### Endpoints

The primary endpoint was the recurrence of ATa; this was defined as a documented episode of AF, LA flutter or LA tachycardia (whether symptomatic or not) lasting for 30 s or more on any cardiac rhythm recording made during a planned or symptom-driven consultation after the three-month blanking period. Electrophysiologically confirmed recurrences of right atrial flutter were not included because they are easier to treat (i.e. with higher success rates and fewer complications) than left atrial arrhythmia.

The secondary endpoint was the frequency of complications of any type during the procedure or in the month thereafter.

### Statistical Analysis

Continuous variables were expressed as the mean ± standard deviation (SD) or the median [interquartile range (IQR)] for normally and non-normally distributed data, respectively, and were compared using Student's *t*-test or the Mann–Whitney–Wilcoxon test, as appropriate. Categorical variables were expressed as the frequency (percentage) and were compared using a chi-squared test or Fisher's exact test.

The cumulative probability of ATa-free survival was estimated using the Kaplan-Meier method. Survival curves were compared using the log-rank test.

Cox proportional hazard models were used to identify factors associated with ATa recurrence, with an estimation of the hazard ratio (HR) and its 95% confidence interval (CI). Variables with *p* < 0.10 in the univariate analysis were included in a multivariate Cox model.

All tests were two-tailed, and the threshold for statistical significance was set to *p* < 0.05. All statistical analyses were performed with SPSS for Windows software (version 22.0, SPSS Inc., Chicago, IL, USA).

The only missing data concerned the LA area (28%), the overall duration of the procedure (8%), the balloon dwell time in the LA (6%), the fluoroscopy time (11%), the fluoroscopy dose (14%) and the duration of the cryo-PVI procedure (8%).

## Results

### Patient Characteristics

**Figure 1:**
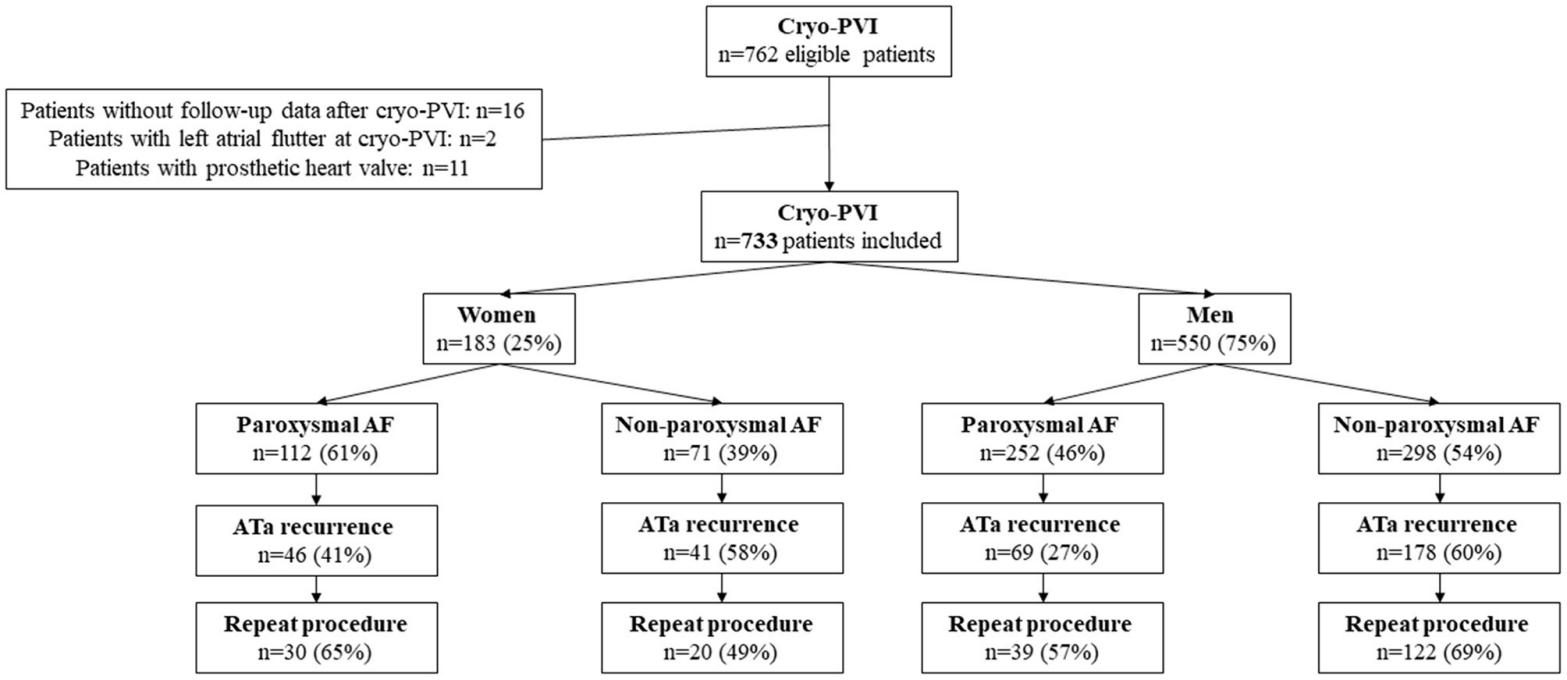
Study flow chart.

A total of 762 patients met the study's inclusion criteria. Sixteen patients were excluded because they had been lost to follow-up after cryo-PVI, and two were excluded because they were in LA flutter at the cryo-PVI. Eleven patients with a prosthetic heart valve were also excluded. Hence, 733 patients (550 male patients (75%) and 183 (25%) female patients) were included in the final analysis (Figure 1).

Paroxysmal AF was observed in 112 (61%) female patients and 252 (46%) male patients (*p* < 0.001). On average, the female patients were older than the male patients at the time of the cryo-PVI procedure (mean ± SD age: 63 ± 9 vs. 60 ± 10, respectively; *p* < 0.001). The diagnosis-to-ablation time was similar in men and women, regardless of whether they suffered from paroxysmal or non-paroxysmal AF (Table 1). Female patients had a greater symptom burden at baseline (mean European Heart Rhythm Association (EHRA) score: 2.3 ± 0.5, vs. 2.2 ± 0.4 in male patients; *p* = 0.04). Female patients had a lower mean creatinine clearance rate (78 ± 21, vs. 83 ± 21 mL/min in male patients; *p* = 0.002) and were more likely to have hypothyroidism (17%, vs. 8% in male patients; *p* = 0.001). Structural heart disease was less common in women than in men (19% vs. 31%, respectively; *p* = 0.001). The LA area and LA volume were significantly smaller in female patients than in male patients (respectively 23 ± 5 vs. 25 ± 5 cm^2^ for the mean LA area, and 133 ± 45 vs. 149 ± 45 ml for the mean LA volume; *p* < 0.001 for both).

**Table 1:** Characteristics of the study population.

	**All patients *n* = 733**	**Men** ***n* = 550 (75%)**	**Women** ***n* = 183 (25%)**	***p***
**Demographic variables**				
Age (years)	61 ± 10	60 ± 10	63 ± 9	**<0.001**
**Medical history**				
Height (m)	1.74 ± 0.1	1.78 ± 0.1	1.65 ± 0.1	**<0.001**
Weight (kg)	87 ± 17	90 ± 16	78 ± 16	**<0.001**
Body mass index (kg/m^2^)	29 ± 5	29 ± 5	29 ± 6	0.81
Body surface area (m^2^)	2.07 ± 0.2	2.12 ± 0.2	1.91 ± 0.2	**<0.001**
Cardioversion	0.8 ± 0.9	0.8 ± 0.9	0.8 ± 1	0.96
Number of failed antiarrhythmic drugs	1.1 ± 0.7	1.1 ± 0.7	1.1 ± 0.8	0.87
Creatinine clearance rate (ml/min)	82 ± 21	83 ± 21	78 ± 21	**0.002**
Hypertension, n (%)	338 (46)	254 (46)	84 (46)	1
Diabetes, n (%)	85 (12)	66 (12)	19 (10)	0.60
Stroke, n (%)	49 (7)	33 (6)	16 (9)	0.23
Hypothyroidism, n (%)	73 (10)	42 (8)	31 (17)	**0.001**
Heart failure, n (%)	97 (13)	72 (13)	25 (14)	0.90
Structural heart disease, n (%)	204 (28)	170 (31)	34 (19)	**0.001**
Coronary artery disease, n (%)	87 (12)	82 (15)	5 (3)	**<0.001**
Oral anticoagulation therapy				
DOA, n (%)	549 (75)	410 (74)	139 (76)	0.77
VKA, n (%)	184 (25)	140 (26)	44 (24)	
CHA_2_DS_2_-VASc score, n (%)	1.7 ± 1.4	1.4 ± 1.3	2.5 ± 1.2	
0	175 (24)	175 (32)	0 (0)	
1	201 (27)	158 (29)	44 (24)	
2	159 (22)	110 (20)	49 (27)	
3	114 (16)	58 (10)	56 (31)	**<0.001**
4	61 (8)	36 (6)	25 (14)	
5	16 (2)	10 (2)	6 (3)	
6	6 (1)	4 (1)	2 (1)	
7	1 (0.1)	0 (0)	1 (0.5)	
EHRA score, n (%)	2.2 ± 0.4	2.2 ± 0.4	2.3 ± 0.5	**0.04**
I	5 (1)	4 (1)	1 (1)	
II	506 (77)	395 (79)	111 (70)	
III	148 (22)	102 (20)	46 (29)	
IV	1 (0.2)	1 (0.2)	0 (0)	
Previous CTI ablation, n (%)	71 (10)	59 (11)	12 (7)	0.11
**AF characteristics**				
Paroxysmal AF, n (%)	364 (50)	252 (46)	112 (61)	**<0.001**
Non-paroxysmal AF, n (%)	369 (50)	298 (54)	71 (39)	
Diagnosis-to-ablation time (months)				
Paroxysmal AF	46 ± 54	48 ± 58	40 ± 44	0.14
Non-paroxysmal AF	19 ± 20	19 ± 21	15 ± 18	0.06
**LA and LV parameters**				
LA area (cm^2^)	24 ± 5	25 ± 5	23 ± 5	**<0.001**
LA volume (ml)	144 ± 45	149 ± 45	133 ± 45	**<0.001**
LA volume index (ml/m^2^)	70 ± 21	70 ± 20	70 ± 24	0.93
LVEF (%)	57 ± 11	56 ± 11	58 ± 10	0.13
LVEF ≤ 40%, n (%)	90 (12)	72 (13)	18 (10)	0.30
**PV anatomy**	
LSPV diameter (mm)	19 ± 2	19 ± 2	18 ± 2	**<0.001**
LIPV diameter (mm)	17 ± 2	17 ± 2	16 ± 3	**<0.001**
RSPV diameter (mm)	19 ± 2	19 ± 2	18 ± 2	**<0.001**
RIPV diameter (mm)	18 ± 2	18 ± 2	17 ± 2	**<0.001**
LCT, n (%)	106 (14)	78 (14)	28 (15)	0.72
Accessory veins	96 (13)	73 (13)	23 (13)	0.90

*AF, atrial fibrillation; CTI, cavotricuspid isthmus; DOA, direct oral anticoagulant; LA, left atrium; LCT, left common trunk of PV; LIPV, left inferior pulmonary vein; LSPV, left superior pulmonary vein; LVEF, left ventricular ejection fraction; LV, left ventricle; PV, pulmonary vein; RIPV, right inferior pulmonary vein; RSPV, right superior pulmonary vein; VKA, vitamin K antagonist*.

*The bold values indicate the values of p < 0.05*.

One hundred and sixty one (64%) of the male patients with paroxysmal AF and 58 (52%) of the female patients with paroxysmal AF had been treated with one or more antiarrhythmic drugs before the cryo-PVI. One hundred and eighty six (62%) of the male patients with non-paroxysmal AF and 38 (54%) of the female patients with non-paroxysmal AF had been treated with one or more antiarrhythmic drugs before the cryo-PVI.

### Characteristics of the Cryo-PVI Procedure

The 23 mm-cryoballoon was used in 27 (15%) female patients and 20 (4%) male patients (Supplementary Table 1). The trough temperature was lower in male patients than in female patients. We did not observe sex differences in the time to effect, the number of cryoballoon applications, the overall procedure time, the balloon dwell time in the LA, or the fluoroscopy time. The mean fluoroscopy dose was lower in female patients (126 ± 188, vs. 189 ± 283 mGy in male patients; *p* = 0.002).

### Complications

Female patients were more likely to experience complications (*p* = 0.02) in general and stroke (1.6%, vs. 0.2% in male patients, *p* = 0.05) and pericardial effusion/tamponade (1.6%, vs. 0.2% in male patients, *p* = 0.05) in particular (Table 2).

**Table 2:** Complications.

	**All patients** ***n* = 733**	**Men** ***n* = 550 (75%)**	**Women** ***n* = 183 (25%)**	***p***
Complications	38 (5.2)	22 (3.0)	16 (8.7)	**0.02**
phrenic nerve palsy	22 (3.0)	15 (2.7)	7 (3.8)	0.46
stroke	4 (0.5)	1 (0.2)	3 (1.6)	**0.05**
vascular complication	6 (0.8)	3 (0.5)	3 (1.6)	0.17
pericardial effusion/tamponade	4 (0.5)	1 (0.2)	3 (1.6)	**0.05**
gastroparesis	2 (0.3)	2 (0.4)	0	1
Outcome after phrenic nerve palsy				
recovery after ≤ 1 month	11 (50)	8 (53)	3 (42)	
recovery after 2 to 6 months	3 (14)	3 (20)	0	0.12
recovery after 7 to 12 months	6 (27)	4 (27)	2 (29)	
persistence of palsy	2 (9)	0	2 (29)	

*The bold values indicate the values of p < 0.05*.

The three strokes in female patients were due to sylvian artery occlusion, whereas the male patient had experienced a multifocal stroke. One patient had speech sequelae. Three women experienced pericardial effusion without the need for drainage. One male experienced cardiac tamponade, which resolved after percutaneous drainage. The vascular complications comprised four cases (three women and one man) with hematoma (with no need for transfusion), a man with arteriovenous fistulae (treated successfully with a covered stent), and a man with a pseudoaneurysm, which disappeared spontaneously. Among the 22 patients with phrenic nerve palsy, the recovery time was <1 month for 11 (50%), between 2 and 6 months for three (14%), and between 7 and 12 months for six (27%). Two patients with phrenic nerve palsy (both women) did not recover: one is asymptomatic and the other required surgery.

### Follow-Up

Seven hundred and twenty-five patients (99%) completed at least 12 months of follow-up. The median length of follow-up was 31 months (IQR 18–50) for the study population as a whole, 32 months (IQR 18–52) for male patients, and 29 months (IQR 18–46) for female patients (*p* = 0.22). The mean ± SD time to recurrence was 26 ± 21 months in male patients and 23 ± 19 months in female patients (*p* = 0.13).

### Outcome After Cryo-PVI for Paroxysmal AF, and Predictors of ATa Recurrence

**Figure 2:**
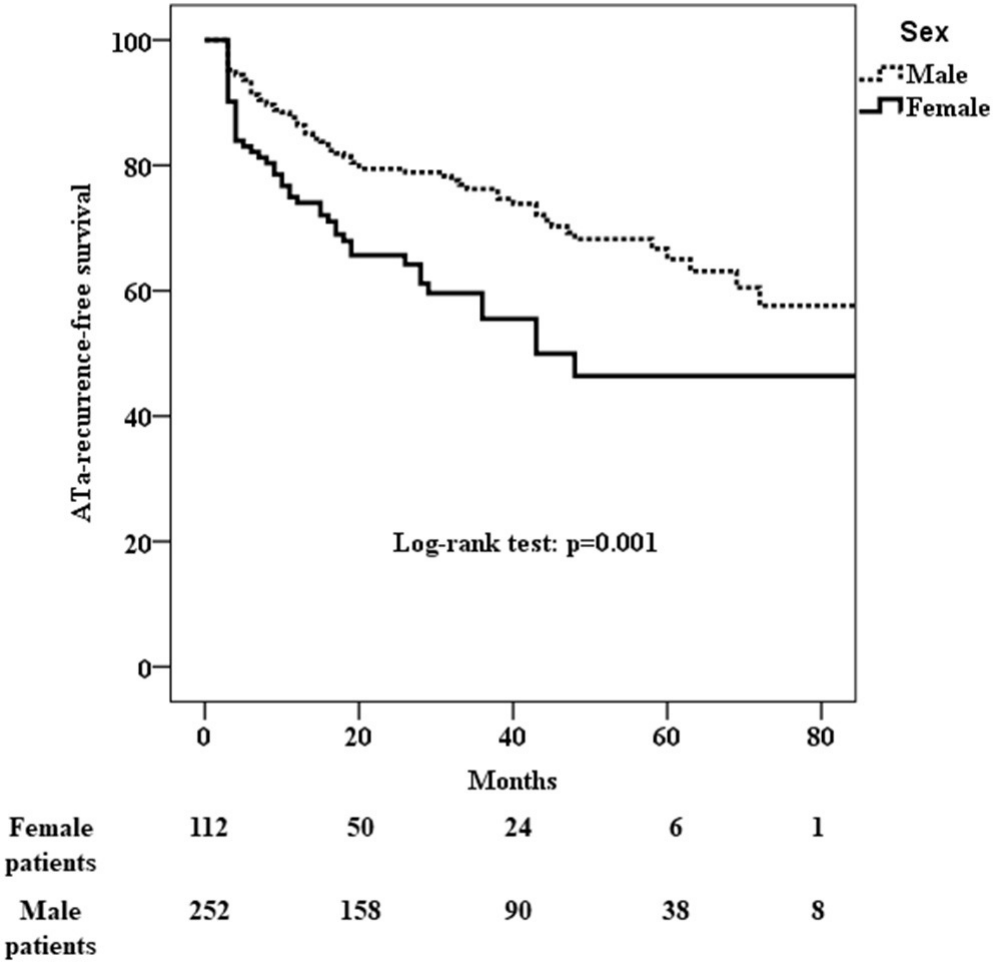
Kaplan-Meier analysis of cumulative survival for ATa recurrence after cryo-PVI for paroxysmal AF. Female patients had a significantly higher risk of recurrence (*p* = 0.001).

ATa recurred in 46 (41%) of the 112 female patients and in 69 (27%) of the 252 male patients who had undergone cryo-PVI for paroxysmal AF (Table 3). The Kaplan-Meier estimate of the ATa-free survival rate at 24 months was 66% [61–70%] for female patients and 79% [77–82%] for male patients. Female patients had a significantly higher risk of ATa recurrence after an index cryo-PVI for paroxysmal AF (*p* = 0.001) (Figure 2). In a univariate analysis, female sex (HR [95%CI] =1.84 [1.26; 2.68]; *p* = 0.002) and height (HR [95%CI] = 0.05 [0.006; 0.39]; *p* = 0.004) were predictors of ATa recurrence (Supplementary Table 2). In a multivariate analysis, female sex (HR [95%CI] = 1.87 [1.28; 2.73]; *p* = 0.001) was the only predictor of ATa recurrence (Table 4). The LA volume index was close to being statistically significant (HR [95%CI] = 1.011 [1.00; 1.02]; *p* = 0.052).

**Table 3:** Sex differences in outcomes after an index cryo-PVI for paroxysmal AF.

**Paroxysmal AF at the time of cryo-PVI**	**Men** ***N* = 252**	**Women** ***N* = 112**	***p***
ATa recurrence, n (%)	69 (27)	46 (41)	**0.01**
Type of recurrence, n (%)			
paroxysmal AF	50 (72)	32 (70)	
non-paroxysmal AF	11 (16)	10 (22)	0.68
left atrial flutter	8 (12)	4 (8)	
Kaplan-Meier estimation of the ATa-free survival rate, %			
24 months	79 [77–82]	66 [61–70]	0.001^*^
36 months	76 [73–79]	55 [50–61]	

*AF, atrial fibrillation; ATa, atrial tachyarrhythmia; PVI; pulmonary vein isolation*.

* *In a log-rank test*.

*The bold values indicate the values of p < 0.05*.

**Table 4:** Predictive factors (multivariate analysis) of ATa recurrence after an index cryo-PVI for paroxysmal AF.

	**Paroxysmal AF** ***n*** **=** **364**	
	**HR [95%CI] in a multivariate analysis**	***p***
Female sex, n (%)	**1.87 [1.28; 2.73]**	**0.001**
Height (m)	0.16 [0.01; 2.24]	0.17
Body surface area (m^2^)	0.99 [0.33; 2.91]	0.98
Structural heart disease, n (%)	0.74 [0.44; 1.24]	0.26
LA volume index (ml/m^2^)	1.01 [1.00; 1.02]	0.052

*AF, atrial fibrillation; ATa, atrial tachyarrhythmia; LA, left atrium*.

*The bold values indicate the values of p < 0.05*.

### Outcome After Cryo-PVI for Non-paroxysmal AF, and Predictors of ATa Recurrence

**Figure 3:**
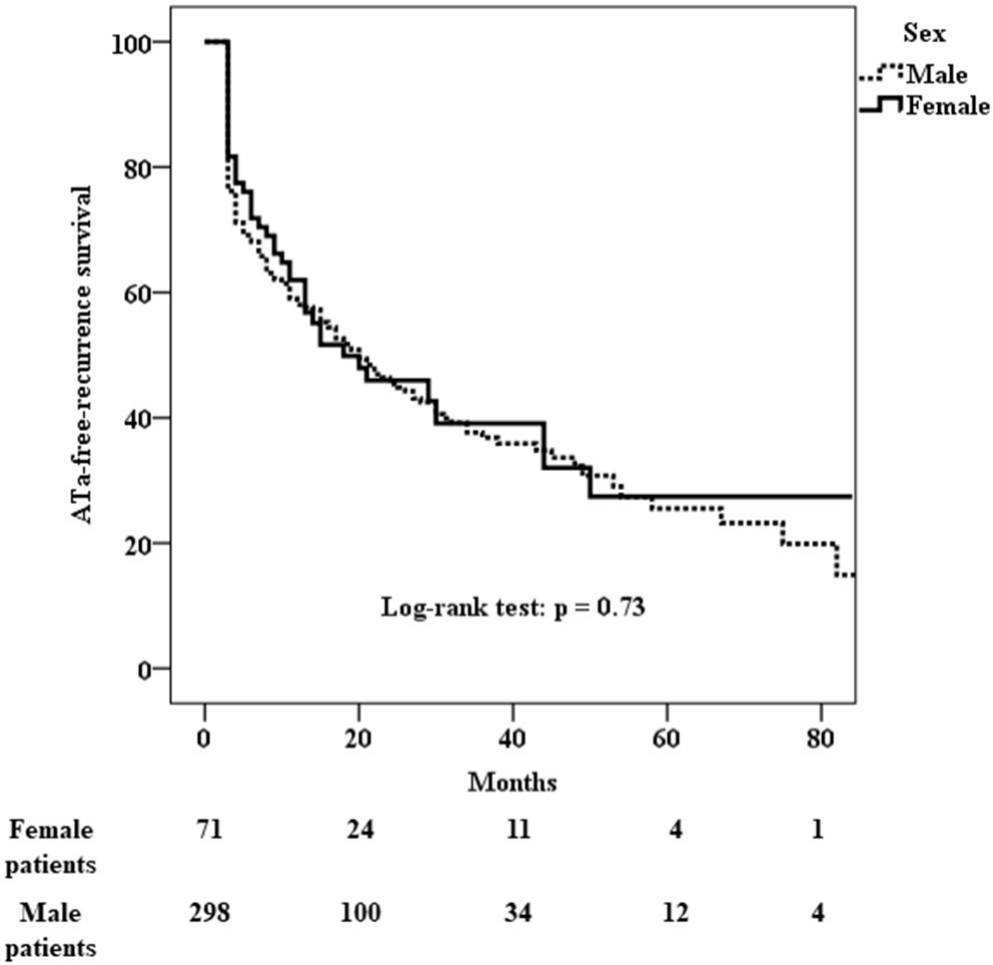
Kaplan-Meier analysis of cumulative survival for ATa recurrence after cryo-PVI for non-paroxysmal AF. There was no sex difference in the risk of recurrence (*p* = 0.73).

ATa recurred in 41 (60%) of the 71 female patients and in 178 (60%) of the 298 male patients who underwent cryo-PVI for non-paroxysmal AF (Table 5). The Kaplan-Meier estimate of the ATa-free survival rate at 24 months was 46% [40–52%] for female patients and 45% [42–48%] for male patients. There was no difference in the risk of ATa recurrence between male and female patients with non-paroxysmal AF (*p* = 0.73) (Figure 3). The predictors of ATa recurrence in a univariate analysis are shown in Supplementary Table 3. In a multivariate analysis (Table 6), body mass index (HR [95%CI] = 1.03 [1.003; 1.07]; *p* = 0.03), long-standing persistent AF (HR [95%CI] = 2.03 [1.38;2.98]; p < 0.001), accessory veins (HR [95%CI] = 1.67 [1.06; 2.61]; *p* = 0.03), and the LA volume index (HR [95%CI] = 1.01 [1.007; 1.02]; *p* < 0.001) were found to be predictors of ATa recurrence.

**Table 5:** Sex differences in outcomes after an index cryo-PVI for non-paroxysmal AF.

**Non-paroxysmal AF at the time of cryo-PVI**	**Men** ***N* = 298**	**Women** ***N* = 71**	***p***
ATa recurrence, n (%)	178 (60)	41 (58)	0.79
Type of recurrence, n (%)			
paroxysmal AF	26 (15)	9 (22)	
non-paroxysmal AF	127 (71)	27 (66)	0.51
left atrial flutter	25 (14)	5 (12)	
Kaplan-Meier estimation of the ATa-free survival rate, %			
24 months	45 [42–48]	46 [40–52]	0.73^*^
36 months	37 [33–40]	39 [32–46]	

*AF, atrial fibrillation; ATa, atrial tachyarrhythmia; PVI, pulmonary vein isolation*.

* *In a log-rank test*.

**Table 6:** Predictive factors (multivariate analysis) of ATa recurrence after index cryo-PVI for non-paroxysmal AF.

	**Patients with non-paroxysmal AF** ***n*** **=** **369**	
	**HR [95%CI] in a multivariate analysis**	***p***
Body mass index (kg/m^2^)	**1.03 [1.003; 1.07]**	**0.03**
Long-standing persistent AF, n (%)	**2.03 [1.38; 2.98]**	**<0.001**
Hypertension, n (%)	1.18 [0.86;1.61]	0.30
LA area (cm^2^)	1.03 [0.99;1.08]	0.19
LA volume (ml)	0.99 [0.98;1.01]	0.37
LA volume index (ml/m^2^)	**1.01 [1.007; 1.02]**	**<0.001**
Accessory vein, n (%)	**1.67 [1.06; 2.61]**	**0.03**

*AF, atrial fibrillation; ATa, atrial tachyarrhythmia; LA, left atrium*.

*The bold values indicate the values of p < 0.05*.

### Outcomes After Repeat Procedures

Of the 46 female patients and 69 male patients with ATa recurrence after cryo-PVI for paroxysmal AF, respectively 30 (65%) and 39 (57%) underwent at least one repeat procedure (*p* = 0.44). We found at least one reconnected PV at the first repeat procedure in 24 (80%) female patients and 29 (74%) male patients (*p* = 0.77). The mean number of reconnected PVs was 1.9 ± 1.2 in female patients and 1.8 ± 1.2 in male patients (*p* = 0.75). The right inferior PV was the most frequently reconnected PV in both female patients (53%) and male patients (56%, *p* = 0.81, Supplementary Table 4). The mean number of repeat procedures was 1.3 ± 0.5 in female patients and 1.2 ± 0.5 in male patients (*p* = 0.12). After the last procedure, 88 (79%) of the female patients and 215 (85%) of the male patients were in sinus rhythm (*p* = 0.13). At last follow-up after an index cryo-PVI for paroxysmal AF, 69 male patients (27%) and 27 (24%) female patients were still taking antiarrhythmic drugs (*p* = 0.61).

Of the 41 female patients and 178 male patients with ATa recurrence after cryo-PVI for non-paroxysmal AF, respectively 20 (49%) and 122 (69%) underwent at least one repeat procedure (*p* = 0.019). We found at least one reconnected PV at the first repeat procedure in 12 (60%) female patients and 64 (52%) male patients (*p* = 0.63). The mean number of reconnected PVs at the first repeat procedure was 1.0 ± 1.0 in female patients and 0.9 ± 1.0 in male patients (*p* = 0.62). The mean number of procedures was 1.3±0.5 in female patients and 1.5 ± 0.7 in male patients (*p* = 0.002). After the last procedure, 42 (59%) female patients and 210 (70%) male patients were in sinus rhythm (*p* = 0.09). At last follow-up after an index cryo-PVI for non-paroxysmal AF, there was no difference in the persistence of antiarrhythmic drug treatment between male patients and female patients (32 vs. 31%, respectively; *p* = 1).

The reasons for non-performance of a repeat procedure after the first ATa recurrence (123 patients) were similar in men and women (*p* = 0.56): the electrophysiologist's decision for 60 male patients (70%) and 23 female patients (62%), the patient's decision for 12 male patients (14%) and eight female patients (22%), and non-referral to the ablation center for 14 male patients (16%) and six female patients (16%).

## Discussion

Our present results showed that relative to male patients, female patients were more likely to be in paroxysmal AF. Although the women were older, they were not referred to an ablation center later than men. We also found that the index cryo-PVI for paroxysmal AF was significantly less successful in women than in men, and that the complication rate was slightly higher in women than in men. Lastly, female patients with non-paroxysmal AF were less likely to undergo a repeat procedure after ATa recurrence.

### Patient Characteristics

In cases of paroxysmal AF, the proportion of patients undergoing cryo-PVI was higher for women than for men (61% vs. 46%, respectively; *p* < 0.001); this difference is often reported in the literature (7, 11, 12, 20, 24, 25, 29). Female patients had a greater symptom burden at baseline (mean EHRA scores: 2.3 ± 0.5, vs. 2.2 ± 0.4 in men; *p* = 0.04), as also extensively reported in the literature (5–12, 25). This difference might be due to the higher average heart rate in women (31). Furthermore, women with AF were more likely to experience longer symptomatic episodes and more frequent recurrences of AF (32). We also found that female patients were older than male patients at the time of the cryo-PVI (63 ± 9 vs. 60 ± 10, respectively; *p* < 0.001), as observed previously (4, 11–14, 16–20, 22–25, 29). Population-based studies have demonstrated that on average, new-onset AF starts 5 years later in women than in men (33). It has been suggested that sex-dependent physiological and pathophysiological mechanisms predispose men to AF earlier in life. In our study, however, the diagnosis-to-ablation-time was similar in the two sexes; this suggest that women were not referred later than men in our study—in contrast to two former studies (13, 14). However, our results are in line with the recent reports by Ricciardi et al. (11) and Singh et al. (22); this might indicate a change in practice over time. Female patients had a significantly lower mean creatinine clearance rate [*p* = 0.001, as in other studies (19, 29)] and were more likely to suffer from hypothyroidism [*p* = 0.001, as in the study by Bukari et al. (29)]. In contrast, structural heart disease was less prevalent in women (20% vs. 31% in men; *p* = 0.005), as was less coronary artery disease (*p* < 0.001); these differences are well-known (12, 14, 16, 17, 19, 20, 23, 29).

### Outcomes of Cryo-PVI

In the present study, female patients had a higher risk of recurrence after an index cryo-PVI for paroxysmal AF (*p* = 0.001), and female sex was the only independent predictor of ATa recurrence—even though the LA volume index was close to being statistically significant. In Kuck et al.'s study (19) (which included patients with paroxysmal AF), female sex was also independently associated with the recurrence of atrial arrhythmia. Many other studies [including studies of non-paroxysmal AF, and particular Cheung et al.'s study of 54,597 patients (16)] found that the success rate for cryo-ablation was lower in women than in men (11, 14, 20) and that female sex was an independent predictor of AF recurrence (11, 14). In a meta-analysis published in 2019, Cheng et al. also confirmed that the recurrence rate was higher in women (34). In our study, women were not referred later for ablation and did not have a greater comorbidity burden. Consequently, this variable could not explain the sex difference in the ablation success rate. Several studies have shown that non-PV triggers are more frequent in women than in men (14, 35); this might explain the lower ablation success rate. This hypothesis is strengthened by the fact that after eliminating non-PV foci, Takigawa et al. (23) did not observed the same sex differences as we did. In the present study, the cryo-PVI trough temperature was lower in male patients than in female patients; this might have enabled the creation of longer-lasting lesions. Alternatively, the sex difference might be due to more frequent consultation for symptomatic recurrence by women than by men. Lastly, sex hormones might have a role in the progression of AF. In the CIRCA-DOSE sub study (36) that used implantable monitors, no difference was found in men vs. women who underwent cryo-PVI for paroxysmal AF. This difference could be explained by our protocol of atrial arrhythmia monitoring which does not include continuous monitoring.

In contrast, we did not find a sex difference in the outcome of the index cryo-PVI for non-paroxysmal AF. A study (22) that looked at patients with persistent AF gave the same result. The other studies included all types of AF, with a high proportion of patients with paroxysmal AF; this difference prevents valid interstudy comparisons.

### Complications of Cryo-PVI

The results of large studies have demonstrated clearly that female patients suffer more than male patients from cardiac perforation/effusion/tamponade (5, 15–18, 34). Our findings are in line with the literature data, even though we observed few complications. The incidence of pericardial effusion/tamponade was slightly higher among women than among men (*p* = 0.05). Kaiser et al. (17) hypothesized that differences in LA size and geometry increase the risk of perforation.

We also found that female patients were more likely to suffer from peri-operative stroke (*p* = 0.05). A similar trend was observed in the large studies performed by Kaiser et al. (17) (*p* = 0.09) and Elayi et al. (18) (*p* = 0.07). Likewise, Cheng et al.'s meta-analysis found that women had an greater risk of stroke or transient ischemic attack (34). Female sex is a well-known independent risk factor for thromboembolism (37).

In the present study, the male vs. female difference in the incidence of bleeding/vascular complications was not statistically significant. However, the literature data (5, 14–19) show clearly that these complications are more frequent among women. Sex differences in vascular anatomy and anticoagulation responses may explain the elevated incidence of vascular complications in women.

### Repeat Procedures

For patients with ATa recurrence after cryo-PVI for paroxysmal AF, women underwent repeat procedures as often as male patients did (*p* = 0.12); this probably explains why the overall success rate at last follow-up in women (79% in sinus rhythm) was as good as in men (85%; *p* = 0.13). However, female patients in non-paroxysmal AF at the time of the index cryo-PVI were less likely to undergo a repeat procedure than male patients (*p* = 0.002). This difference might explain the non-significant trend toward a lower success rate for women at last follow-up (59% of the female patients and 70% of the male patients were in sinus rhythm; *p* = 0.09) despite the absence of a sex difference after the index cryo-PVI procedure. Winkle et al. (28) made the same observation; the overall success rate was lower for women because they underwent fewer repeat ablations. Kaiser et al. (17) also found that women were less likely to undergo repeat ablation. In the present study, female patients did not refuse the repeat procedure more than male patients; the difference in the repeat ablation rate was mostly due to the electrophysiologist's decision.

In our repeat procedures, the mean number of reconnected PVs was similar in men and women. In contrast, Sugumar et al. (27) found that lasting PV isolation was significantly more frequent in women than in men (*p* = 0.01); however, this difference might have been be due to Sugumar et al.'s use of radiofrequency ablation rather than cryo-PVI.

### Limitations

Firstly, this was a single-center study, and so its conclusions cannot be extrapolated to other settings. Secondly, echocardiographic measurement of the LA area is subject to interobserver variability and so conclusions based on this variable must be considered with caution. However, these readily available data are rarely mentioned in the literature. CT volume measurement is a new parameter and might be less subject to less interobserver variability. The absence of long-term monitoring (a loop recorder or 7-day Holter) means that the frequency of atrial arrhythmia recurrence and asymptomatic episodes might have been underestimated.

## Conclusion

Relative to men, women referred for cryo-PVI were more likely to be in paroxysmal AF. Furthermore, the women were older, more symptomatic at the time of the index cryo-PVI procedure, and more likely to experience periprocedural complications. For patients with paroxysmal AF (but not those with non-paroxysmal AF), female sex was the only independent predictor of recurrence after cryo-PVI. Repeat ablation was less common in female patients treated for non-paroxysmal AF.

## Data Availability Statement

The raw data supporting the conclusions of this article will be made available by the authors, without undue reservation.

## Ethics Statement

The studies involving human participants were reviewed and approved by CNIL PI2020_843_0080. The patients/participants provided their written informed consent to participate in this study.

## Author Contributions

AH, JB, MK, FF, P-ML, OB, AL, MZ, CB, and J-SH contributed to conception and design of the study. AH, JB, FF, AL, MZ, and J-SH organized the database. AH performed the statistical analysis and wrote the first draft of the manuscript. AH and J-SH wrote sections of the manuscript. All authors contributed to manuscript revision, read, and approved the submitted version.

## Conflict of Interest

The authors declare that the research was conducted in the absence of any commercial or financial relationships that could be construed as a potential conflict of interest.

## Publisher's Note

All claims expressed in this article are solely those of the authors and do not necessarily represent those of their affiliated organizations, or those of the publisher, the editors and the reviewers. Any product that may be evaluated in this article, or claim that may be made by its manufacturer, is not guaranteed or endorsed by the publisher.

## Abbreviations

AF: atrial fibrillation
Ata: atrial tachyarrhythmia
Cryo-PVI: cryoballoon pulmonary vein isolation
LA: left atrial
LVEF: left ventricular ejection fraction
HR: hazard ratio.
