# Supplementary material for: Sex Differences in the Outcomes of Cryoablation for Atrial Fibrillation
Source: Front Cardiovasc Med. 2022 May 18;9:893553. doi: 10.3389/fcvm.2022.893553 (PMC9157614; doi:10.3389/fcvm.2022.893553)
Supplement: Supplementary file 1 [file Data_Sheet_1.DOCX]

Supplementary table 1. Characteristics of the ablation procedure.

|  | **All patients**  n=733 | **Men**  n=550 (75%) | **Women**  n=183 (25%) | **p** |
| --- | --- | --- | --- | --- |
| Sinus rhythm at the time of the procedure, n (%) | 417 (57) | 298 (54) | 119 (65) | **0.02** |
| 23 mm, n (%) | 47 (6) | 20 (4) | 27 (15) | **<0.001** |
| 28 mm, n (%) | 669 (91) | 520 (94) | 149 (81) |  |
| 23+28 mm, n (%) | 17 (2) | 10 (2) | 7 (4) |  |
| Advance short tip, n (%) | 109 (15) | 87 (16) | 22 (12) |  |
| LSPV min T° (°C) | -50 ± 7 | -50 ± 7 | -48 ± 7 | **0.008** |
| LIPV min T° | -46 ± 7 | -46 ± 7 | -45 ± 8 | 0.07 |
| RSPV min T° | -50 ± 7 | -51 ± 7 | -48 ± 7 | **<0.001** |
| RIPV min T° | -48 ± 8 | - 49 ± 8 | -47 ± 7 | **0.05** |
| LSPV cryo time (s) | 316 ± 139 | 311 ± 136 | 331 ± 147 | 0.10 |
| LIPV cryo time | 311 ± 133 | 308 ± 134 | 317 ± 131 | 0.48 |
| RSPV cryo time | 283 ± 133 | 281 ± 133 | 291 ± 133 | 0.36 |
| RIPV cryo time | 301 ± 144 | 303 ± 143 | 294 ± 148 | 0.47 |
| LSPV TTE (s) | 64 ± 43 | 64 ± 42 | 65 ± 46 | 0.91 |
| LIPV TTE | 60 ± 41 | 59 ± 40 | 64 ± 44 | 0.34 |
| RSPV TTE | 44 ± 36 | 43 ± 35 | 50 ± 41 | 0.16 |
| RIPV TTE | 65 ± 48 | 67 ± 47 | 59 ± 53 | 0.34 |
| LSPV block, n (%) | 732 (99) | 550 (100) | 182 (99) | 0.25 |
| LIPV block | 733 (100) | 550 (100) | 183 (100) |  |
| RSPV block | 727 (99) | 546 (99) | 181 (99) | 0.64 |
| RIPV block | 723 (99) | 545 (99) | 178 (97) | 0.13 |
| LSPV block first shoot, n (%) | 483 (66) | 368 (67) | 115 (63) | 0.32 |
| LIPV block first shoot | 501 (68) | 384 (70) | 117 (64) | 0.14 |
| RSPV block first shoot | 535 (73) | 407 (74) | 128 (70) | 0.29 |
| RIPV block first shoot | 525 (72) | 396 (72) | 129 (71) | 0.71 |
| LSPV applications | 1.4 ± 0.8 | 1.4 ± 0.8 | 1.4 ± 0.7 | 0.81 |
| LIPV applications | 1.4 ± 0.7 | 1.4 ± 0.6 | 1.4 ± 0.7 | 0.13 |
| RSPV applications | 1.3 ± 0.7 | 1.3 ± 0.6 | 1.4 ± 0.8 | 0.13 |
| RIPV applications | 1.4 ± 0.7 | 1.4 ± 0.7 | 1.3 ± 0.7 | 0.61 |
| Total n applications | 5.5 ± 1.6 | 5.4 ± 1.5 | 5.6 ± 1.9 | 0.41 |
| CTI at PVI, n (%) | 147 (20) | 111 (20) | 36 (20) | 0.92 |
| Balloon in/out (min) | 42 ± 13 | 42 ± 13 | 42 ± 13 | 0.89 |
| Fluoroscopy time (min) | 14 ± 8 | 14 ± 8 | 15 ± 9 | 0.10 |
| Fluoroscopy dose (mGy) | 174 ± 265 | 189 ± 283 | 126 ± 188 | **0.002** |
| Fluoroscopy dose (Gy.cm²) | 31±83 | 35±95 | 22±37 | 0.29 |
| Total cryo time (min) | 1334 ± 412 | 1327 ± 400 | 1358 ± 447 | 0.41 |
| Total procedure time (min) | 83 ± 23 | 83 ± 22 | 83 ± 25 | 0.64 |

AF: atrial fibrillation, CTI: cavotricuspid isthmus ablation, LIPV: left inferior pulmonary vein, LSPV: left superior pulmonary vein, PVI: pulmonary vein isolation, RIPV: right inferior pulmonary vein, RSPV: right superior pulmonary vein, TTE: time to effect
