# Supplementary material for: Sex Differences in the Outcomes of Cryoablation for Atrial Fibrillation
Source: Front Cardiovasc Med. 2022 May 18;9:893553. doi: 10.3389/fcvm.2022.893553 (PMC9157614; doi:10.3389/fcvm.2022.893553)
Supplement: Supplementary file 2 [file Data_Sheet_2.DOCX]

|  | **Paroxysmal AF**  n = 364 | | | |
| --- | --- | --- | --- | --- |
|  | **No ATa recurrence**  249 (68) | **ATa recurrence**  115(32) | **HR [95%CI] in a univariate analysis** | **p** |
| Age (y) | 61 ±10 | 61±11 | 0.99 [0.98;1.02] | 0.83 |
| Female sex, n (%) | 66 (26) | 46 (40) | **1.84 [1.26;2.68]** | **0.002** |
| Height (m) | 1.74±0.09 | 1.71±0.09 | **0.05 [0.006;0.39]** | **0.004** |
| Weight (kg) | 83±15 | 81±14 | 0.99 [0.98;1.003] | 0.14 |
| Body mass index (kg/m^2^) | 27±5 | 28±4 | 1.003 [0.96;1.04] | 0.89 |
| Body surface area (m^2^) | 2.02±0.2 | 1.99±0.2 | 0.43 [0.17;1.08] | 0.07 |
| Diagnosis-to-ablation-time (months) | 44±56 | 50±50 | 1.001 [0.99;1.004] | 0.50 |
| Creatinine clearance rate (ml/min) | 83±21 | 81±21 | 0.99 [0.99;1.003] | 0.23 |
| Hypertension, n (%) | 110 (44) | 49 (43) | 0.89 [0.62;1.29] | 0.55 |
| Diabetes, n (%) | 24 (10) | 12 (10) | 0.99 [0.54;1.80] | 0.97 |
| Heart failure, n (%) | 17 (7) | 12 (10) | 1.27 [0.70;2.32] | 0.43 |
| Coronary artery disease, n (%) | 33 (13) | 8 (7) | 0.48 [0.23;0.98] | 0.044 |
| Structural heart disease, n (%) | 52 (21) | 18 (16) | 0.66 [0.40;1.09] | 0.10 |
| CHA_2_DS_2_-VASc score | 1.6±1.3 | 1.9±1.5 | 1.10 [0.97;1.25] | 0.15 |
| Previous CTI, n (%) | 23 (9) | 18 (16) | 1.54 [0.93;2.56] | 0.09 |
| LA area (cm^2^) | 22±5 | 22±5 | 1.02 [0.98;1.06] | 0.42 |
| LA volume (ml) | 121±33 | 125±38 | 1.003 [0.99;1.01] | 0.28 |
| LA volume index (ml/m^2^) | 60±16 | 63±19 | 1.01 [1.0;1.02] | 0.06 |
| LVEF (%) | 60±8 | 60±8 | 1.00 [0.98;1.02] | 0.97 |
| LVEF <50%, n (%) | 19 (8) | 8 (7) | 0.92 [0.45;1.89] | 0.82 |
| LVEF ≤40%, n (%) | 14 (6) | 7 (6) | 1.08 [0.50;2.32] | 0.84 |
| Left common trunk, n (%) | 32 (13) | 18 (16) | 1.20 [0.72;1.99] | 0.47 |
| Accessory vein, n (%) | 28 (11) | 21 (18) | 1.31 [0.81;2.11] | 0.26 |

Supplementary table 2. Predictive factors (univariate analysis) of ATa recurrence after an index cryo-PVI for paroxysmal AF

AF: atrial fibrillation, ATa: atrial tachyarrhythmia, CTI: cavotricuspid isthmus ablation, LA: left atrium, LV: left ventricle, LVEF: left ventricle ejection fraction.
