# Supplementary material for: Sex Differences in the Outcomes of Cryoablation for Atrial Fibrillation
Source: Front Cardiovasc Med. 2022 May 18;9:893553. doi: 10.3389/fcvm.2022.893553 (PMC9157614; doi:10.3389/fcvm.2022.893553)
Supplement: Supplementary file 3 [file Data_Sheet_3.DOCX]

Supplementary table 3. Predictive factors (univariate analysis) of ATa recurrence after an index cryo-PVI for non-paroxysmal AF

|  | **Patients with non-paroxysmal AF**  n = 369 | | | |
| --- | --- | --- | --- | --- |
|  | **No ATa recurrence**  n= 150 (41%) | **ATa recurrence**  n= 219 (59%) | **HR [95%CI] in a univariate analysis** | **p** |
| Age (y) | 60±10 | 60±9 | 1.006 [0.99;1.02] | 0.43 |
| Female sex, n (%) | 30 (20) | 41 (19) | 0.94 [0.67;1.33] | 0.74 |
| Height (m) | 1.76±0.1 | 1.75±0.1 | 0.91 [0.21;3.96] | 0.90 |
| Weight (kg) | 89±18 | 93±17 | 1.006 [0.99;1.01] | 0.11 |
| Body mass index (kg/m^2^) | 29±5 | 30±5 | **1.03 [1.002;1.05]** | **0.03** |
| Body surface area (m^2^) | 2.10±0.3 | 2.15±0.2 | 1.50 [0.84;2.69] | 0.17 |
| Diagnosis-to-ablation-time (months) | 17±20 | 20±21 | 1.005 [0.99;1.01] | 0.11 |
| Long-standing persistent AF, n (%) | 10 (7) | 50 (23) | **2.23 [1.62;3.07]** | **<0.001** |
| Creatinine clearance rate (ml/min) | 81±21 | 82±22 | 0.99 [0.99;1.005] | 0.77 |
| Hypertension, n (%) | 65 (43) | 114 (52) | 1.30 [0.99;1.70] | 0.053 |
| Diabetes, n (%) | 18 (12) | 31 (14) | 1.27 [0.87;1.86] | 0.21 |
| Heart failure, n (%) | 34 (23) | 34 (15) | 0.75 [0.52;1.09] | 0.13 |
| Coronary artery disease, n (%) | 13 (9) | 33 (15) | 1.34 [0.93;1.95] | 0.12 |
| Structural heart disease, n (%) | 51 (34) | 83 (38) | 1.10 [0.84;1.45] | 0.49 |
| CHA_2_DS_2_-VASc score | 1.6 ±1.4 | 1.7±1.4 | 1.08 [0.98;1.19] | 0.11 |
| Previous cavotricuspid isthmus ablation, n (%) | 13 (9) | 17 (8) | 0.97 [0.59;1.59] | 0.89 |
| LA area (cm^2^) | 25±4 | 27±5 | **1.08 [1.04;1.11]** | **<0.001** |
| LA volume (ml) | 156±42 | 175±43 | **1.01 [1.005;1.01]** | **<0.001** |
| LA volume Index (ml/m^2^) | 74 ±19 | 82±22 | **1.01 [1.008;1.02]** | **<0.001** |
| LVEF (%) | 54±12 | 54±12 | 0.99 [0.99;1.01] | 0.88 |
| LVEF <50%, n (%) | 43 (29) | 48 (22) | 0.87 [0.63;1.21] | 0.42 |
| LVEF ≤40%, n (%) | 31 (21) | 38 (17) | 0.97 [0.68;1.37] | 0.85 |
| Left common trunk, n (%) | 23 (15) | 33 (15) | 0.94 [0.65;1.37] | 0.76 |
| Accessory vein, n (%) | 14 (9) | 33 (15) | 1.46 [1.009;2.12] | 0.045 |

AF: atrial fibrillation, ATa: atrial tachyarrhythmia, LA: left atrium, LV: left ventricle, LVEF: left ventricle ejection fraction.
