# Supplementary material for: Sex Differences in the Outcomes of Cryoablation for Atrial Fibrillation
Source: Front Cardiovasc Med. 2022 May 18;9:893553. doi: 10.3389/fcvm.2022.893553 (PMC9157614; doi:10.3389/fcvm.2022.893553)
Supplement: Supplementary file 4 [file Data_Sheet_4.DOCX]

Supplementary table 4. Data about reconnected PVs at the first repeat procedure

|  | Repeat procedures after an index cryo-PVI for **paroxysmal AF**  n=69 | | | Repeat procedures after an index cryo-PVI for **non**-**paroxysmal AF**  n=142 | | |
| --- | --- | --- | --- | --- | --- | --- |
|  | **Men**  n=39 | **Women**  n=30 | **p** | **Men**  n=122 | **Women**  n=20 | **p** |
| LSPV reconnection | 14 (36) | 10 (33) | 1 | 21 (17) | 3 (15) | 1 |
| LIPV reconnection | 18 (46) | 14 (47) | 1 | 25 (20) | 8 (40) | 0.08 |
| RSPV reconnection | 14 (36) | 16 (53) | 0.22 | 23 (19) | 3 (15) | 1 |
| RIPV reconnection | 22 (56) | 16 (53) | 0.81 | 38 (31) | 5 (25) | 0.79 |

AF: atrial fibrillation. LSPV: left superior pulmonary vein. LIPV: left inferior pulmonary vein. V: pulmonary vein. PVI: pulmonary vein isolation. RSPV: right superior pulmonary vein. RIPV: right inferior pulmonary vein. P
